# ResMap: A community resource for systematic mapping of therapy-persistent residual cancer cell dependencies across contexts

**DOI:** 10.1126/sciadv.aed7476

**Published:** 2026-06-12

**Authors:** Xiaoxiao Sun, Savitha Gayathri, Karl Kumbier, Heinz Hammerlindl, Erin Ahern, Lani F. Wu, Steven J. Altschuler

**Affiliations:** Department of Pharmaceutical Chemistry, University of California, San Francisco, CA, USA.

## Abstract

Relapse following targeted therapy remains a central challenge in oncogene-driven cancers. Drug-tolerant persister cells that survive initial treatment without genetic resistance seed relapse, yet despite over a decade of research, no persister-directed therapy has reached clinical approval. To bridge this gap, we developed ResMap, a community resource providing both a standardized experimental framework and quantitative dataset for systematic comparison of persister vulnerabilities across cancer contexts. Using this platform, we evaluated 94 compounds targeting 57 literature-derived candidates in two *EGFR*^*mut*^ and two *KRAS*^*G12C*^ lung cancer models under normoxic and hypoxic conditions. Initial screening identified 12 targets with conserved anti-persister activity across genotypes and oxygen environments; follow-up validation reproduced 9 of these targets and revealed variable degrees of persister specificity relative to general cytotoxicity. We also identified context-specific vulnerabilities, including KRAS-relevant combination targets, several of which have since been independently reported and/or advanced to clinical testing. Integration of human cancer cell line essentiality data and adult mouse loss-of-function phenotypes provided a complementary tolerability layer to refine prioritization. ResMap establishes a foundation for coordinated community efforts to accelerate rational persister-directed combination strategies toward the clinic.

## Teaser

A standardized framework compares, validates, and prioritizes cancer persister targets.

## INTRODUCTION

Residual disease following targeted therapy remains a major obstacle to durable responses in oncogene-driven cancers (*1*). Drug-tolerant persister cells—subpopulations that survive initial therapy without stable genetic resistance—can contribute to residual disease and seed tumor relapse (*2*, *3*). Understanding and targeting these cells have emerged as a promising strategy for achieving lasting therapeutic outcomes.

Since the first description of cancer cell “persistence” in 2010 (*4*), studies across cancer types have linked persister survival to diverse biological processes, including metabolic reprogramming, epigenetic remodeling, DNA damage tolerance, and stress response activation (*5*–*8*). These efforts have produced an expanding list of candidate therapeutic targets.

However, differences in experimental models, treatment contexts, perturbation methods, and assay readouts limit direct comparison and hinder systematic prioritization. Moreover, key microenvironmental variables, including oxygen tension, are rarely incorporated into persister studies despite their potential effects on cellular state and drug response. How do reported persister targets compare under a standardized framework? Which targets are broadly conserved versus context-specific across genetic, treatment, and environmental contexts?

To address these questions, we developed ResMap (Residual Disease Map), a standardized framework that integrates automated persister assays, image-based quantification, persistence-specific readout, and machine learning normalization. We curated community-identified persister targets, spanning multiple cancer types and diverse cellular processes, and assembled a benchmark compound library for rapid functional screening. As a testbed, we chose lung cancer (where most curated persister targets were originally reported), two oncogenic driver contexts (*EGFR*^*mut*^, with many targets identified, and *KRAS*^*G12C*^, with only a few reported targets), and two oxygen tensions to capture environmental variation. This design yielded ~10,000 persister measurements.

Our goals for ResMap are to provide (i) a curated, community-informed persister target and compound library for functional evaluation; (ii) a standardized and extensible experimental and computational framework enabling direct cross-context comparison; (iii) a validation layer that refines target prioritization through reproducibility, persister specificity, and genetic tolerability data; and (iv) a quantitative reference dataset distinguishing conserved from context-specific persister targets. ResMap provides a community resource for coordinated validation efforts and rational combination design aimed at minimizing residual disease following anticancer therapy.

## RESULTS

### ResMap: Survey of persister targets and benchmark compound library

We surveyed the persister literature to establish the foundation for our systematic evaluation. Since the initial description of persisters in 2010 (*4*), PubMed-indexed persister publications have increased steadily (fig. S1A), underscoring the rapid growth of the field and the need for cross-comparable datasets.

**Fig. 1.**
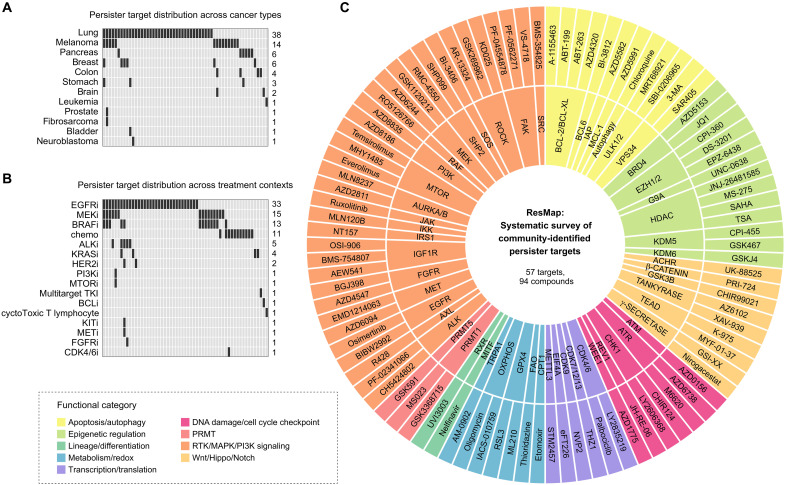
ResMap survey of community-identified persister targets and benchmark compound library for systematic evaluation. (**A** and **B**) Distribution of curated persister targets across cancer types (A) and treatment contexts (B). Each column represents one target. Total counts of targets are shown on the right. (**C**) Composition of the ResMap benchmark library with 57 community-identified persister targets (inner ring) and 94 associated compounds (outer ring), color labeled by functional category.

From this body of community research (through 2022), we curated 57 persister targets reported to influence persister survival (table S1). Mapping these targets across cancer types and treatment contexts revealed a strong concentration in lung cancer and receptor tyrosine kinase (RTK)/mitogen-activated protein kinase (MAPK) axis–targeted therapy contexts (Fig. 1, A and B). This pattern reflects both the historical origin and translational focus of the persister field: Persisters were first characterized in *EGFR*^*mut*^ lung cancer models treated with epidermal growth factor receptor (EGFR)–targeted therapy, which many subsequent studies adopted. Moreover, precision medicine in lung cancer, where inhibitors of EGFR, ALK (anaplastic lymphoma kinase), and other RTK/MAPK pathway components produce high initial response rates, has brought the challenge of residual disease into focus. Meanwhile, reports across other cancer types and treatment contexts, including chemotherapy and immunotherapy, demonstrate that persistence represents a broadly conserved biological phenomenon.

These community-identified targets span diverse functional categories (Fig. 1C and table S1): RTK/MAPK/phosphatidylinositol 3-kinase (PI3K) signaling [e.g., EGFR, FGFR (fibroblast growth factor receptor), AXL, SHP2 (Src homology 2 domain–containing protein tyrosine phosphatase 2), RAF, and MEK (MAPK kinase)], apoptosis/autophagy [BCL-2 (B cell lymphoma 2)/BCL-XL, MCL-1 (myeloid cell leukemia 1), IAP (inhibitor of apoptosis protein), ULK1/2 (Unc-51–like autophagy-activating kinase 1 and 2), and VPS34 (vacuolar protein sorting 34 homolog)], epigenetic regulation [BRD4 (bromodomain containing 4), EZH1/2 (enhancer of zeste homolog 1 and 2), G9A, KDM5/6 (lysine demethylase 5 and 6), and HDAC (histone deacetylase)], Wnt/Hippo/Notch [β-CATENIN, GSK3B (glycogen synthase kinase 3 beta), TANKYRASE, TEAD (TEA domain transcription factor), and γ-SECRETASE], DNA damage/checkpoint [ATM (ataxia-telangiectasia mutated), ATR (ataxia telangiectasia and Rad3-related protein), CHK1 (checkpoint kinase 1), WEE1, and REV1], transcription/translation [CDK7/9/12/13 (cyclin-dependent kinase 7/9/12/13), EIF4A (eukaryotic translation initiation factor 4A), and METTL3 (N6-adenosine-methyltransferase 70 kDa subunit)], metabolism/redox [OXPHOS (oxidative phosphorylation), GPX4 (glutathione peroxidase 4), FAO (fatty acid oxidation), and CPT1 (carnitine palmitoyltransferase 1)], lineage/differentiation [RXR (retinoid X receptor) and MITF (microphthalmia-associated transcription factor)], and protein methyltransferases (PRMTs), underscoring the mechanistic diversity of processes capable of sustaining persister survival.

To rapidly and systematically evaluate these targets across a large number of persister contexts, we compiled a corresponding set of 94 compounds, generating the ResMap benchmark compound library (Fig. 1C and table S2). Compounds were selected on the basis of reported potency and selectivity to minimize off-target effects and ensure interpretable functional readouts. Most targets were represented by one or two compounds, whereas several heavily studied targets [e.g., BCL-2/BCL-XL, IGF1R (insulin-like growth factor 1 receptor), and HDAC] had broader compound coverage (fig. S1B). This library enables functional testing of diverse community-nominated persister targets.

### ResMap: Standardized framework for persister perturbation screening

**Fig. 2.**
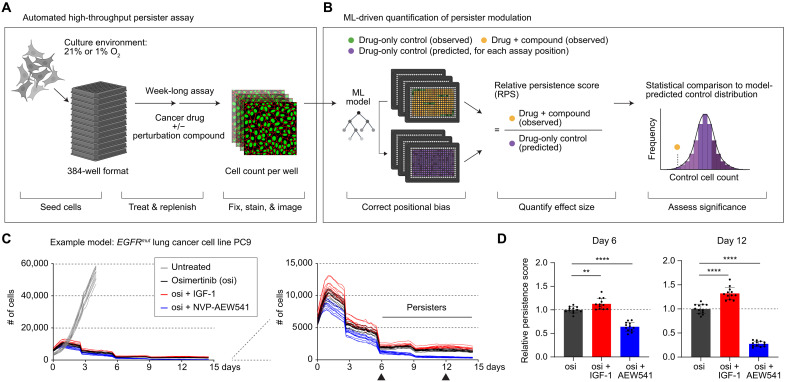
Development of the ResMap platform for standardized persister perturbation screening. (**A**) Automated 384-well workflow integrating liquid handling, periodic medium replenishment, controlled oxygen environments, and image-based cell counting over week-long assays. (**B**) Machine learning (ML)–based normalization using spatially distributed drug-only controls to correct for plate-position effects. Perturbation significance is calculated relative to model-predicted control distribution. See fig. S1 for model evaluation and Materials and Methods for detailed screening procedures. (**C**) Conventional viability metrics normalized to untreated controls obscure changes in small surviving persister populations. Representative time course of *EGFR*^*mut*^ PC9 cells under indicated conditions: untreated (gray), EGFR inhibitor osimertinib at 100 nM (black), osimertinib + IGF-1 at 20 ng/ml (red), and osimertinib + IGF1R inhibitor NVP-AEW541 at 100 nM (blue). Curves show initial cytotoxicity followed by a persister plateau. Periodic drops reflect debris removal during medium changes. (**D**) Validation of the RPS [RPS = cell count (drug + perturbation)/cell count (drug only)]. IGF-1 increases RPS (pro-persistence), whereas AEW541 decreases RPS (anti-persistence). Bars represent the means ± standard deviation across 12 replicate wells after 6 days (left) or 12 days (right) of treatment. **P ≤ 0.01 and ****P ≤ 0.0001, unpaired two-sided Student’s *t* tests.

To enable rigorous cross-context comparison and systematic prioritization, we developed the ResMap platform incorporating four integrated components: an automated high-throughput workflow, machine learning–based normalization, a persistence-specific metric, and a validation framework. First, we established an automated 384-well workflow integrating liquid handling, controlled oxygen environments, periodic medium replenishment, and image-based cell counting over week-long assays (Fig. 2A and Materials and Methods). This automation enabled the scale needed for systematic persister evaluation while maintaining assay consistency. Second, we implemented machine learning–based normalization using random forest models trained on spatially distributed drug-only controls to correct for plate-position effects and enable statistical assessment of perturbation significance (Fig. 2B, fig. S2A, and Materials and Methods). Third, we developed the relative persistence score (RPS) (*9*, *10*), a metric specifically designed to quantify changes in small persister populations that standard viability metrics can obscure (Fig. 2, C and D). The RPS normalizes perturbation compound–treated persister abundance to cancer drug–only controls [RPS = cell count (drug + compound)/cell count (drug only)], providing a sensitive readout for both anti-persistence (RPS < 1) and pro-persistence (RPS > 1) effects. Fourth, we established a validation workflow to test reproducibility and assess persister specificity by comparing compound activity in drug-treated persisters versus untreated parental cells (Materials and Methods).

As a testbed, we selected four lung cancer models: two with EGFR inhibitor osimertinib (EGFRi)–treated *EGFR*^*mut*^ cell lines (PC9 and MGH134) and two with KRAS inhibitor sotorasib (KRASi)–treated *KRAS*^*G12C*^ cell lines (LU65 and MGH1138-1). *EGFR*^*mut*^ lung cancer remains the most extensively studied model system in the field, where 33 of our 57 curated persister targets were originally identified (table S1). In contrast, *KRAS*^*G12C*^ lung cancer is a newly targetable subtype where approved inhibitors achieve only transient responses (*11*–*13*). At the time of study initiation, relatively few persister targets had been reported in this context, making it a timely setting for systematic benchmarking.

To capture potential effects of oxygen tension, we screened each model under both normoxic (21% O_2_) and severe hypoxic (1% O_2_) conditions. Each compound was tested at three concentrations spanning low, medium, and high dose ranges on the basis of reported potency and prior cellular usage (medians, 0.17, 0.67, and 2 μM, respectively; fig. S2B and table S2), with triplicate measurements per condition. We chose a 6-day time point to measure persistence to accommodate the feasibility for a large-scale screen yet still maintain the ability to identify the persister-modulating effect (Fig. 2D). This experimental design generated ~10,000 RPS measurements (ResMap benchmark dataset; table S3), providing a robust, internally controlled foundation for cross-condition analysis.

### ResMap: Compound activity landscape

To systematically identify active compounds, for each compound and dose, we computed the median RPS across triplicates. Compounds are classified as active if the median RPS < 0.5 (*P* < 0.05) at any tested dose (fig. S3A). This criterion identifies compounds that reproducibly reduce persister abundance by at least 50% relative to drug-only controls. Across all tested compounds, 67 of 94 (71.3%) were active in at least one ResMap condition, and each condition contained between 29 (30.9%) and 53 (56.4%) active compounds (Table 1 and fig. S3B).

**Table 1.** Compound-level activity in the ResMap screen. The table summarizes the number and percentage (in parentheses) of active compounds across conditions. A compound was classified as active if it met both criteria (median RPS < 0.5 and *P* < 0.05) at any tested dose.

O_2_ condition	Oncogene driver	Cell line	Active per model	Per oncogene driver		Per O_2_ condition		Overall	
				Overlap	Union	Overlap	Union	Overlap	Union
21% O_2_	*KRAS* ^*G12C*^	LU65	36 (38.30%)	32 (34.04%)	52 (55.32%)	16 (17.02%)	61 (64.89%)	14 (14.89%)	67 (71.28%)
		MGH1138-1	48 (51.06%)						
	*EGFR* ^*mut*^	PC9	44 (46.81%)	26 (27.66%)	50 (53.19%)				
		MGH134	32 (34.04%)						
1% O_2_	*KRAS* ^*G12C*^	LU65	39 (41.49%)	30 (31.91%)	45 (47.87%)	18 (19.15%)	60 (63.83%)		
		MGH1138-1	36 (38.30%)						
	*EGFR* ^*mut*^	PC9	53 (56.38%)	27 (28.72%)	55 (58.51%)				
		MGH134	29 (30.85%)						

**Fig. 3.**
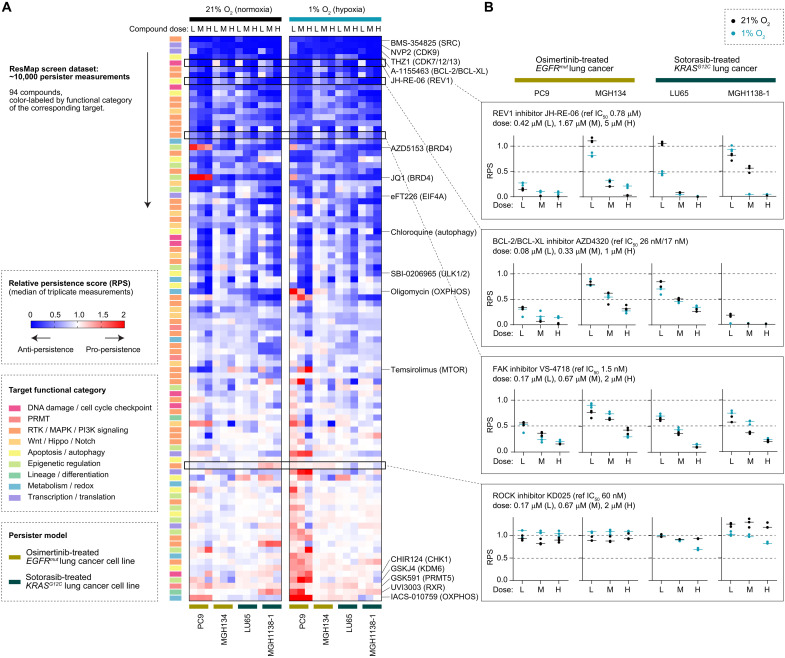
Compound activities from the ResMap screen. (**A**) Composition and global compound-level activity of the ResMap dataset (~10,000 persister measurements from 4 lung cancer models × 2 oxygen levels × 3 compound doses × 3 replicates). Four lung cancer models (two *EGFR*^*mut*^ and two *KRAS*^*G12C*^) were cotreated with the indicated cancer drug (100 nM osimertinib or 1 μM sotorasib, respectively) and test compound for 6 days. Screening was performed under two oxygen conditions (21% and 1% O_2_) and three compound doses [low (L), medium (M), and high (H)]. The heatmap displays median RPS values from triplicate measurements for 94 compounds across all conditions. Each row represents one compound, ranked by mean RPS across doses, models, and oxygen levels. Blue indicates the anti-persistence effect (RPS < 1), and red indicates the pro-persistence effect (RPS > 1). Functional target categories are annotated on the left. (**B**) Representative compound responses showing triplicate measurements (dots) and median values (solid lines). Top panels: The REV1 inhibitor JH-RE-06 and the BCL-2/BCL-XL inhibitor AZD4320 demonstrate dose-dependent anti-persistence activity with varying magnitudes across models. Middle panel: The FAK inhibitor VS-4718 shows similar graded anti-persistence activities across models. Bottom panel: The ROCK inhibitor KD025 shows no detectable anti-persistence activity. Reported IC_50_ (median inhibitory concentration) values (cell-free assays) are indicated in parentheses for comparison with effective cellular doses in the ResMap screen. Detailed screening procedures are described in Materials and Methods.

Visualization of the ResMap data revealed broad anti-persistence activity across diverse biological processes (Fig. 3A). Rather than clustering within a single functional category, anti-persistence effects were distributed across multiple processes, reflecting the mechanistic diversity of community-identified targets. Representative examples include transcriptional regulation (the CDK7/12 inhibitor THZ1) (*14*), apoptosis activation (the BCL-2/BCL-XL inhibitor A-1155463) (*3*), and DNA damage tolerance (the REV1 inhibitor JH-RE-06) (*15*). Reassuringly, we observed broad dose-dependent modulation of persistence, in some cases with different magnitudes of response across models (e.g., the REV1 inhibitor JH-RE-06 and the BCL-2/BCL-XL inhibitor AZD4320; Fig. 3B, top panels) and in other cases with similar graded responses across models [e.g., the focal adhesion kinase (FAK) inhibitor VS-4718; Fig. 3B, middle panel]. However, not all compounds elicited responses in our tested models; some showed no detectable activity [e.g., the Rho-associated coiled-coil–containing protein kinase (ROCK) inhibitor KD025; Fig. 3B, bottom panel]. Several compounds even increased persister survival, such as the BRD4 inhibitors AZD5153 and JQ1, which displayed pro-persistence effects specifically in PC9, and the MTOR (mechanistic target of rapamycin) inhibitor temsirolimus, which showed opposing behaviors depending on model and oxygen condition. These observations reveal a rich variety of responses across models and conditions, reflecting specific biological interactions rather than simple additive toxicity.

Among compounds selected to modulate targets originally identified outside of lung cancer, we identified several that demonstrated strong anti-persistence activity in lung cancer models. Notable examples include the CDK9 inhibitor NVP2, first described in chemotherapy-treated or CDK4/6 inhibitor–treated breast cancer persisters (*16*), and the autophagy inhibitors chloroquine and SBI-0206965 (via targeting ULK1/2), originally reported in pancreatic and colorectal cancer persisters (*17*, *18*). We also identified other compounds that showed little or no activity in our lung cancer models, such as the RXR antagonist UVI3003 and the ROCK inhibitor KD025 mentioned above (melanoma) (*19*, *20*), the KDM6 inhibitor GSKJ4 (glioblastoma) (*21*), and the CHK1 inhibitor CHIR124 (pancreatic cancer) (*17*). This heterogeneity in cross-lineage generalization underscores the value of systematic testing frameworks like ResMap in distinguishing broadly conserved from context-specific persister vulnerabilities.

To assess the impact of oxygen tension on compound responses, we compared activities between normoxia (21% O_2_) and hypoxia (1% O_2_). Across all four lung cancer models, compound-level activities were highly correlated between oxygen levels (*R*^2^ values ranging from 0.67 in PC9 to 0.89 in MGH134; fig. S4A). Nevertheless, several compounds exhibited oxygen-dependent differences. For example, the mitochondrial adenosine 5′-triphosphate synthase inhibitor oligomycin showed stronger anti-persistence activity under normoxia, most notably in PC9 cells (fig. S4B), whereas the EIF4A inhibitor eFT226 demonstrated enhanced activity under hypoxia, particularly in PC9 and LU65 (fig. S4C). These findings reveal that oxygen context can modulate compound efficacy, uncovering both oxygen-agnostic vulnerabilities that remain stable across environments and oxygen-sensitive vulnerabilities that emerge only under specific conditions.

Together, these data reveal a diverse landscape of persister-modulating effects that are largely preserved across oxygen contexts while revealing subsets of oxygen-sensitive vulnerabilities. This compound-level activity establishes the foundation for systematic target-level evaluation.

### ResMap: Target activity landscape

**Fig. 4.**
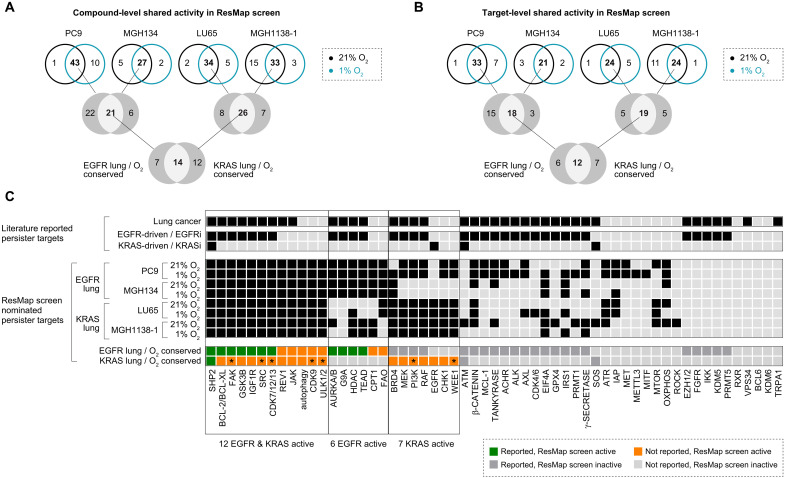
Target-level activities from the ResMap screen. (**A** and **B**) Overlap of active compounds (A) and targets (B) across models under normoxia (black, 21% O_2_) or hypoxia (cyan, 1% O_2_). A compound was classified as active if it met both criteria (median RPS < 0.5 and *P* < 0.05) at any tested dose. A target was considered active if at least one associated compound met these criteria in the ResMap screen. Numbers within circles indicate the number of actives in each condition and their overlaps. (**C**) Target-level activity summary. The upper heatmap shows the cancer types and treatment contexts in which each target was originally reported (black boxes). The middle heatmap displays ResMap target-level activity across four lung cancer models under two oxygen levels (black boxes: active targets). The bottom heatmap summarizes conserved targets within EGFR- or KRAS-driven lung cancer models. The color indicates the relationship to prior literature: green, reported and active in the ResMap screen; orange, novel ResMap screen discovery (* indicates targets independently reported during the course of this study); dark gray, reported but inactive in the ResMap screen; light gray, not previously reported and inactive in the ResMap screen.

We next consolidated compound-level activity (Fig. 4A) into target-level summaries: We assigned activity to a target if at least one corresponding compound met the predefined activity criteria (Fig. 4B). Overall, 47 of 57 (82.5%) targets showed anti-persistence activity in at least one ResMap condition, and each individual condition had between 23 (40.4%) and 40 (70.2%) active targets (Table 2 and fig. S3C).

**Table 2.** Target-level activity in the ResMap screen. The table summarizes the number and percentage (in parentheses) of active targets across conditions. A target was classified as active if at least one associated compound met the compound-level activity criteria.

O_2_ condition	Oncogene driver	Cell line	Active per model	Per oncogene driver		Per O_2_ condition		Overall	
				Overlap	Union	Overlap	Union	Overlap	Union
21% O_2_	*KRAS* ^*G12C*^	LU65	25 (43.86%)	23 (40.35%)	37 (64.91%)	14 (24.56%)	43 (75.44%)	12 (21.05%)	47 (82.46%)
		MGH1138-1	35 (61.40%)						
	*EGFR* ^*mut*^	PC9	34 (59.65%)	21 (36.84%)	37 (64.91%)				
		MGH134	24 (42.10%)						
1% O_2_	*KRAS* ^*G12C*^	LU65	29 (50.88%)	22 (38.60%)	32 (56.14%)	16 (28.07%)	43 (75.44%)		
		MGH1138-1	25 (43.86%)						
	*EGFR* ^*mut*^	PC9	40 (70.18%)	23 (40.35%)	40 (70.18%)				
		MGH134	23 (40.35%)						

To contextualize these findings relative to prior reports, we examined the original discovery settings of each target (table S4). Anti-persistence activity was detected in at least one ResMap condition for 28 of 33 (84.8%) targets first reported in *EGFR*^*mut*^ lung cancer; 3 of 3 (100%) targets (SHP2, SOS, and ATM) first reported in *KRAS*^*G12C*^ lung cancer; and 16 of 19 (84.2%) targets reported in non–lung cancer contexts. These high activity rates support the robustness of many community-reported persister mechanisms and indicate that a substantial fraction of persister targets have generalizable functions across genotypes and experimental systems.

The remaining 10 of 57 (17.5%) targets did not meet our activity threshold under the tested conditions. These included targets reported outside lung cancers [e.g., BCL6 in leukemia (*22*), KDM6 in brain tumor (*21*), and RXR in melanoma (*19*)], as well as several previously reported in lung cancer [e.g., EZH1/2 (*23*), FGFR (*24*), IKK (inhibitor of nuclear factor κB kinase) (*25*), KDM5 (*26*), PRMT5 (*24*, *27*), TRPA1 (transient receptor potential cation channel subfamily A member 1) (*28*), and VPS34 (*29*)].

We then focused on conserved targets—those consistently active across all ResMap screen conditions (Fig. 4C). Notably, 12 targets were active across all models and oxygen conditions (tables S5 and S6), arising from 18 *EGFR*^*mut*^ targets and 19 *KRAS*^*G12C*^ targets active across their respective models at both oxygen levels (table S4). Analysis of these conserved targets revealed activity in “repurposed” contexts. In EGFRi-treated *EGFR*^*mut*^ models, several conserved targets had not been previously reported in this setting (e.g., REV1, CDK9, and ULK1/2). In KRASi-treated *KRAS*^*G12C*^ models, 16 targets emerged as newly identified combination candidates and can be grouped into three evidence tiers: (i) Externally corroborated targets: Several targets [e.g., PI3K (*30*), FAK (*31*), SRC (*32*), CDK12/13 (*33*), CDK9 (*34*), WEE1 (*35*–*37*), and ULK1/2 (*38*)] were independently reported during the course of our study as KRAS combination partners; notably, FAK/SRC and ULK1/2 inhibitors have advanced to clinical testing (NCT06166836, NCT05379946, and NCT04892017). (ii) Cross-lineage support: Other targets [e.g., BRD4 (*39*) and BCL-XL (*40*, *41*)] have been linked to KRAS drug resistance in *KRAS*^*G12D*^ pancreatic or *KRAS*^*G12C*^ colorectal cancers, suggesting generalization across *KRAS* variants and tissue types. (iii) Prospective predictions: Additional targets (e.g., CHK1 and REV1) represent previously unrecognized *KRAS*^*G12C*^ combination hypotheses that warrant independent validation. Together, these conserved targets highlight ResMap’s capacity to reveal both established and emerging persister vulnerabilities, prompting follow-up validation to refine prioritization.

### ResMap: Validation and target prioritization

**Fig. 5.**
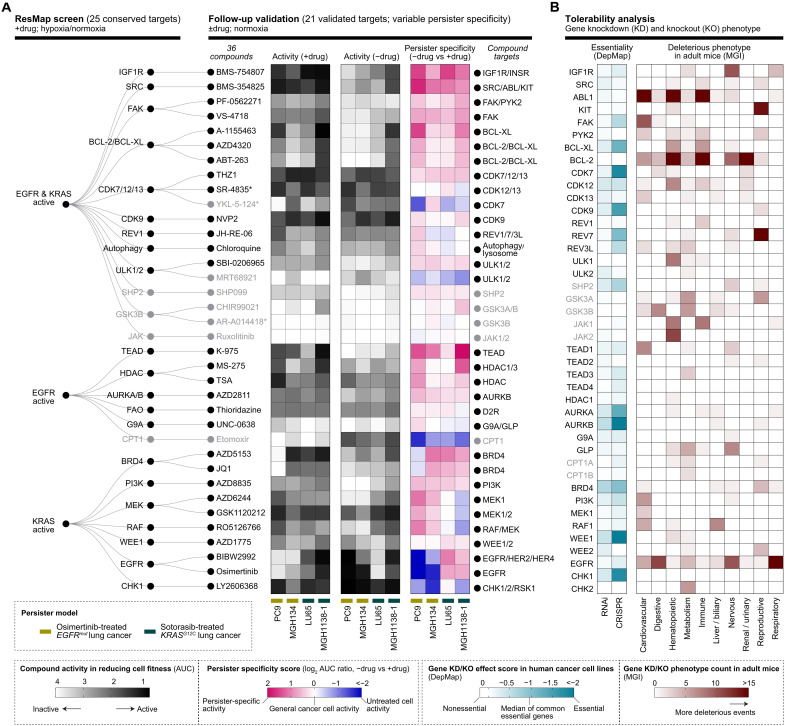
Validation and tolerability assessment of conserved ResMap screen targets. (**A**) Left: Twenty-five priority persister targets nominated from the ResMap screen, grouped by activity pattern (active in both EGFR and KRAS, only EGFR, or only KRAS models). Right: Overview of targets and associated compounds tested in follow-up validation experiments, including original screening compounds and additional inhibitors (*). Four lung cancer cell lines (two *EGFR*^*mut*^ and two *KRAS*^*G12C*^) were treated with the test compound in the presence or absence of the indicated cancer drug (±drug; 100 nM osimertinib or 1 μM sotorasib, respectively) under normoxia for 6 days. The grayscale heatmap shows compound activity quantified as the area under the dose-response curve (AUC), reflecting the overall anti-persistence effect across tested doses; lower AUC values indicate stronger anti-persistence activity. The adjacent two-color heatmap displays persister specificity scores, calculated as log_2_[AUC (−drug)/AUC (+drug)]. Positive values (pink) indicate greater activity in the presence of cancer drug (persister-specific activity), values near zero (white) indicate comparable activity in parental and persister cells, and negative values (blue) indicate greater activity in the absence of cancer drug. Gray text labels: compounds that did not meet activity criteria under +drug condition (median RPS < 0.5 with *P* < 0.05 at any tested dose) and targets where none of the associated compounds met activity criteria. In total, 21 of 25 targets demonstrated reproduced activity. Compound-wise dose-response curves are provided in fig. S5. (**B**) Tolerability analysis of targets. DepMap gene effect scores from RNAi and CRISPR datasets and MGI knockdown (KD) and knockout (KO) phenotype counts (grouped by physiological systems) were integrated to estimate functional essentiality and predicted safety liability. Results across 10 oncology-relevant physiological systems are shown. Full results and detailed analytical details are provided in tables S8 to S10 and Materials and Methods.

Building on the conserved targets identified above, we next performed systematic follow-up experiments to assess reproducibility and refine prioritization. We tested 25 conserved targets emerging from the ResMap screen using all associated compounds from the original dataset, along with additional inhibitors (Fig. 5A, fig. S5, and table S7). Of these, 21 targets demonstrated reproducible anti-persistence activity in the validation experiments. Among the 12 targets initially conserved across both EGFR and KRAS models, 9 retained robust activity upon validation (Fig. 5A, black target/compound text labels). These reproduced conserved persister targets include signaling and adhesion regulators (IGF1R, SRC, and FAK), transcriptional regulators (CDK7/9/12/13), DNA damage tolerance factor (REV1), apoptosis regulators (BCL-2/BCL-XL), and autophagy regulators (ULK1/2).

To further evaluate persister specificity, we tested these targets in the absence of cancer drug across all four lung cancer models. This comparison revealed heterogeneity in specificity: Some targets (e.g., REV1 and CDK9) showed comparable activity in untreated parental or drug-treated persister cells, whereas others (e.g., FAK and SRC) showed enrichment of activity in persister cells (Fig. 5A).

Beyond efficacy, therapeutic prioritization also requires consideration of predicted tolerability. To incorporate this dimension, we integrated Mouse Genome Informatics (MGI) loss-of-function phenotype data and Cancer Dependency Map (DepMap) essentiality data to facilitate evaluations of potential safety liability among top ResMap screen targets (Fig. 5B and tables S8 to S10). Notably, several high-liability targets identified by MGI align with established clinical toxicities, e.g., EGFR-associated respiratory system phenotypes corresponding to interstitial lung disease risk of EGFR inhibitors and BCL-2/BCL-XL–associated hematopoietic system phenotypes corresponding to thrombocytopenia risk of BCL-2/BCL-XL inhibitors, providing concordance between mouse phenotypes and human safety observations. Together, reproducibility testing, persister specificity assessment, and predicted tolerability analysis establish a structured framework for prioritizing persister-directed combination strategies.

## DISCUSSION

Targeting drug-tolerant persister cells has emerged as an essential complement to oncogene-directed therapy, yet the field has lacked a unified framework to evaluate and prioritize candidate targets. The ResMap framework addresses this gap by providing a standardized, extensible platform to test the generality and robustness of reported persister targets under harmonized assay design, readout, and normalization. By applying this framework, we generated the ResMap benchmark dataset, enabling direct, cross-context comparison and facilitating target repurposing across disease settings. ResMap aligns with priorities outlined by the National Cancer Institute Acquired Resistance to Therapy Network (ARTNet) (*42*), which emphasizes reproducible functional assays and shared data standards to accelerate resistance research. Our study establishes ResMap as both a validation framework for community-reported persister targets and discovery platform for context-relevant therapeutic opportunities.

ResMap uncovered a number of context-specific persister vulnerabilities, reflecting the influence of genotype, treatment context, and environmental conditions. Oxygen-sensitive vulnerabilities, particularly those involving mitochondrial metabolism, illustrate how environmental factors can modulate persister responses. For example, OXPHOS inhibitors revealed a reproducible, PC9 model–specific pro-persistence phenotype that was more pronounced under hypoxia, whereas the same inhibitors were relatively more cytotoxic under normoxia. Although the underlying mechanism remains to be determined, this divergence suggests that OXPHOS inhibition may differentially modulate metabolic stress programs depending on cellular state. Similarly, compounds targeting epigenetic regulators such as BRD4 showed divergent, even pro-persistence, effects in select models, emphasizing that not all persister vulnerabilities generalize across contexts. Together, these findings illustrate how systematic cross-context profiling can surface nonintuitive phenotypes that warrant mechanistic investigation, reinforcing the value of a standardized comparative framework, such as ResMap. The heterogeneity observed in ResMap highlights the importance of biomarker-guided strategies to distinguish conserved from context-restricted persister vulnerabilities.

A pattern of conservation also emerged across oxygen conditions, as many persister-modulating effects were preserved under both normoxia and hypoxia. This suggests that the therapy-induced stress response may dominate over oxygen-driven variability in shaping persister vulnerabilities, potentially through suppression of hypoxia-induced signaling (*43*). Although persisters survive through nongenetic mechanisms and might be expected to exhibit highly plastic vulnerabilities, our results suggest that drug exposure can impose a relatively stable stress-adaptive state. Within this induced state, survival programs such as transcriptional control (CDK7/9/12/13) and autophagy regulation (ULK1/2) remain engaged across environmental variation, giving rise to oxygen-agnostic vulnerabilities that represent robust features of persistence and strong candidates for therapeutic combination strategies.

When evaluated within the standardized ResMap framework, 47 of 57 (82.5%) community-nominated persister targets demonstrated anti-persistence activity in at least one condition, supporting the reproducibility of many previously reported persistence mechanisms under harmonized testing. Among these, 12 targets were initially conserved across genotypes and oxygen contexts, and 9 of these retained reproducible activity across EGFR and KRAS models upon follow-up validation. These reproduced conserved targets span signaling and adhesion, transcriptional regulation, apoptosis, autophagy, and DNA damage tolerance pathways. Collectively, these findings suggest that although persister biology involves multiple adaptive programs, targeting individual, well-chosen survival pathways may be sufficient to meaningfully reduce residual disease burden.

Beyond benchmarking, ResMap also demonstrated the capacity for discovery and repurposing. The dataset identified previously underappreciated persister vulnerabilities relevant to KRAS inhibition (e.g., FAK, SRC, CDK7/9/12, REV1, ULK1/2, CHK1, and WEE1), several of which were independently reported or advanced into clinical testing during the course of this study. This underscores how systematic cross-context evaluation can accelerate the translation of emerging vulnerabilities into rational combination strategies.

With the establishment of ResMap, several directions for expansion emerge. First, extending evaluation to additional patient-derived models and cancer types will be essential to define broader generalizability. Second, compound availability and dose selection constraints may have limited detection of some vulnerabilities; future studies incorporating more potent or isoform-selective compounds and complementary genetic perturbations could further refine target assessment. Third, while oxygen variation was included, additional microenvironmental variables (including stromal, immune, and spatial influences) remain unexplored. Fourth, all assays were conducted in vitro; some vulnerabilities observed in ResMap (e.g., IGF1R) have not translated effectively in vivo (*44*), underscoring the need for validation in more physiologically relevant systems. Last, while the current framework captures vulnerabilities involved in persister emergence and early maintenance, extending treatment duration will enable interrogation of later evolutionary and regrowth dynamics under sustained drug pressure.

No single laboratory can comprehensively map persister vulnerabilities across all cancers, treatments, and environmental contexts. The ResMap framework provides a unified foundation for coordinated community efforts. Analogous to large-scale initiatives such as DepMap (https://depmap.org/portal) (*45*), distributed adoption of standardized protocols and shared data infrastructure could enable integrated evaluation across laboratories. Such collective efforts will be required not only to distinguish pan-persister from context-specific vulnerabilities but also to rigorously assess target selectivity across normal tissues, diverse tumor types, treatment modalities, and relapse states. Ultimately, this coordinated approach will guide rational combination strategies aimed at eliminating therapy-persistent residual disease while minimizing toxicity.

## MATERIALS AND METHODS

### Cell lines and reagents

The origins and culture conditions for all cell lines used in this study are detailed in table S11. Cell identities were confirmed by short tandem repeat fingerprinting, and all lines were routinely screened for *Mycoplasma* contamination. Cells were used within 10 to 15 passages postthaw. Additional reagents used are listed in table S12.

#### ResMap compound screen

Cells were maintained under either normoxic (21% O_2_) or hypoxic (1% O_2_) conditions for at least 1 week before screening to allow environmental adaptation. Cells were seeded into 384-well plates at 2000 cells per well (LU65 and MGH134) or 6000 cells per well (PC9 and MGH1138-1). Following attachment, cells were cotreated with the indicated cancer drug (100 nM osimertinib for EGFR models or 1 μM sotorasib for KRAS models) and test compounds for 6 days. Media and drug/compound cotreatment were replenished on day 3. The compound library (table S2) was dispensed using an Echo 650 Acoustic Liquid Handler (Beckman Coulter), and cancer drugs were added using a BioTek MultiFlo FX Multimode Dispenser (Agilent). At the end point, cells were stained with Hoechst 33342 and MitoTracker Deep Red dye for 30 min, fixed with 4% paraformaldehyde, and washed with phosphate-buffered saline using a BioTek 405 LS Microplate Washer (Agilent). Imaging was performed using a PerkinElmer Operetta High Content Imaging System at 10× magnification, capturing nine fields per well. Cell counts were quantified using a segmentation pipeline implemented in Harmony software, generating nuclear (Hoechst) and cytoplasmic (MitoTracker) masks. Quantified cell counts were exported for downstream quantitative analysis.

### Quantitative analysis of ResMap screen measurements

#### Relative persistence normalization

To quantify persistence, we computed an RPS for any given well (*i*), defined as

$$r_{i}=\frac{\text{Observed}\;{\text{cell count}}_{i}}{\text{Corrected control}\;{\text{cell count}}_{i}}$$

The corrected control cell count was estimated using regression models trained on technical variables (plate ID, well row position, and well column position) and O_2_ status. These models allowed us to adjust for position-specific effects and predict drug-only control cell count at noncontrol positions (see the “Model evaluation” section).

#### Model evaluation

We evaluated five approaches to estimate cancer drug–only control cell count:

1) Plate-level average.

2) O_2_ treatment–level average.

3) Linear regression model fit on plate ID + well position (row and column).

4) Linear regression model fit on plate ID + well position + O_2_ condition.

5) Random forest model fit on plate ID + well position + O_2_ condition.

Performance was assessed by the mean absolute error between predicted and observed control viability on a randomly sampled test set (25% of control wells) over 10 bootstrap iterations. Random forests consistently achieved the lowest mean absolute error across cell lines and were used for all subsequent normalization (fig. S2A).

#### Persister modulator identification

For each plate, the statistical significance of a treatment condition was evaluated using a two-sided *t* test comparing relative persistence score distributions between (cancer drug + perturbation compound) and cancer drug–only control replicates.

#### Anti-persistence activity identification

Compounds were classified as active if they met both predefined criteria (median RPS < 0.5 and *P* < 0.05) at any tested dose. Targets were classified as active if at least one associated compound satisfied the compound-level activity criteria in the ResMap screen.

### Persister assays

For temporal monitoring of persister dynamics (Fig. 2, C and D), PC9 cells stably expressing H2B-mCherry were used to enable fluorescence-based quantification of surviving cell numbers. Cells were seeded into 96-well plates, and treatments were initiated on the second day and replenished twice weekly. Longitudinal imaging was performed using the IncuCyte S3 Live-Cell Analysis System (Sartorius). Phase-contrast and red fluorescence images were acquired every 4 hours using a 10× objective, capturing five fields per well. Cell counts were quantified using the integrated IncuCyte analysis software on the basis of the red-channel signal.

Follow-up validation experiments (Fig. 5 and table S7) were performed in 96-well plate format under normoxic conditions (21% O_2_). Cells were seeded at 3000 cells per well for all four lung cancer models. After attachment, cells were treated with either test compound alone (−drug condition) or cotreated with cancer drug and test compound (+drug condition). Treatments were maintained for 6 days, with medium and treatment replenishment on day 3. At the end point, persister fitness was quantified using the CellTiter-Glo luminescent viability assay (Promega) according to the manufacturer’s instructions.

### Tolerability analysis

#### DepMap analysis

To evaluate the functional essentiality of candidate targets nominated in the ResMap screen, we leveraged publicly available gene dependency data from the DepMap. The DepMap provides genome-scale loss-of-function screening results across a large panel of human cancer cell lines using both CRISPR-Cas9 and RNA interference (RNAi) perturbation platforms. We analyzed the top ResMap screen targets. For each gene, gene effect scores were extracted across all available cancer cell lines. The gene effect score reflects the impact of gene knockout or knockdown on cellular fitness and is defined as the log fold change in single guide RNA abundance over time following perturbation. A score of 0 indicates a nonessential gene, whereas −1 corresponds to the median effect observed for common essential genes; increasingly negative values indicate stronger dependency. For each gene, we also calculated the percentage of cancer cell lines with gene effect score <−1. Analyses were performed separately for CRISPR and RNAi datasets to capture complementary measures of functional essentiality (Fig. 5 and table S8).

#### MGI phenotype analysis

Mouse loss-of-function phenotype data for the top ResMap screen target genes were obtained from MGI. Two reports (MGI_PhenotypicAllele.rpt and ALL_Phenotype.rpt) were merged using the MGI Allele Accession ID to link allele attributes with associated phenotype annotations. The dataset was filtered to include loss-of-function allele attribute annotated as “Null/knockout” or “Knockdown.” To focus on adult phenotypes, entries containing the terms “develop,” “embryo,” “prenatal,” “perinatal,” or “neonatal” were excluded. To enable system-level interpretation, phenotypes were categorized into higher-order physiological systems by reconstructing the Mammalian Phenotype (MP) Ontology hierarchy from the MPheno_OBO.ontology file and mapping each phenotype term to its corresponding parent categories. This approach allowed the assignment of phenotypes to defined physiological groups (table S9). For each gene, we quantified the number of associated phenotypes within each physiological group (Fig. 5 and table S10).
