# Supplementary material for: ResMap: A community resource for systematic mapping of therapy-persistent residual cancer cell dependencies across contexts
Source: Sci Adv. 2026 Jun 12;12(24):eaed7476. doi: 10.1126/sciadv.aed7476 (PMC13262613; doi:10.1126/sciadv.aed7476)
Supplement: Supplementary file 1 — Figs. S1 to S5 Legends for tables S1 to S12 [file sciadv.aed7476_sm.pdf]

Science Advances
AAAS

Supplementary Materials for

ResMap: A community resource for systematic mapping of therapy-persistent residual cancer cell dependencies across contexts

Xiaoxiao Sun *et al.*

Corresponding author: Xiaoxiao Sun, xiaoxiao.sun@ucsf.edu; Lani F. Wu, lani.wu@ucsf.edu; Steven J. Altschuler, steven.altschuler@ucsf.edu

Sci. Adv. **12**, eaed7476 (2026)
DOI: 10.1126/sciadv.aed7476

The PDF file includes:

Figs. S1 to S5
Legends for tables S1 to S12

Other Supplementary Material for this manuscript includes the following:

Tables S1 to S12

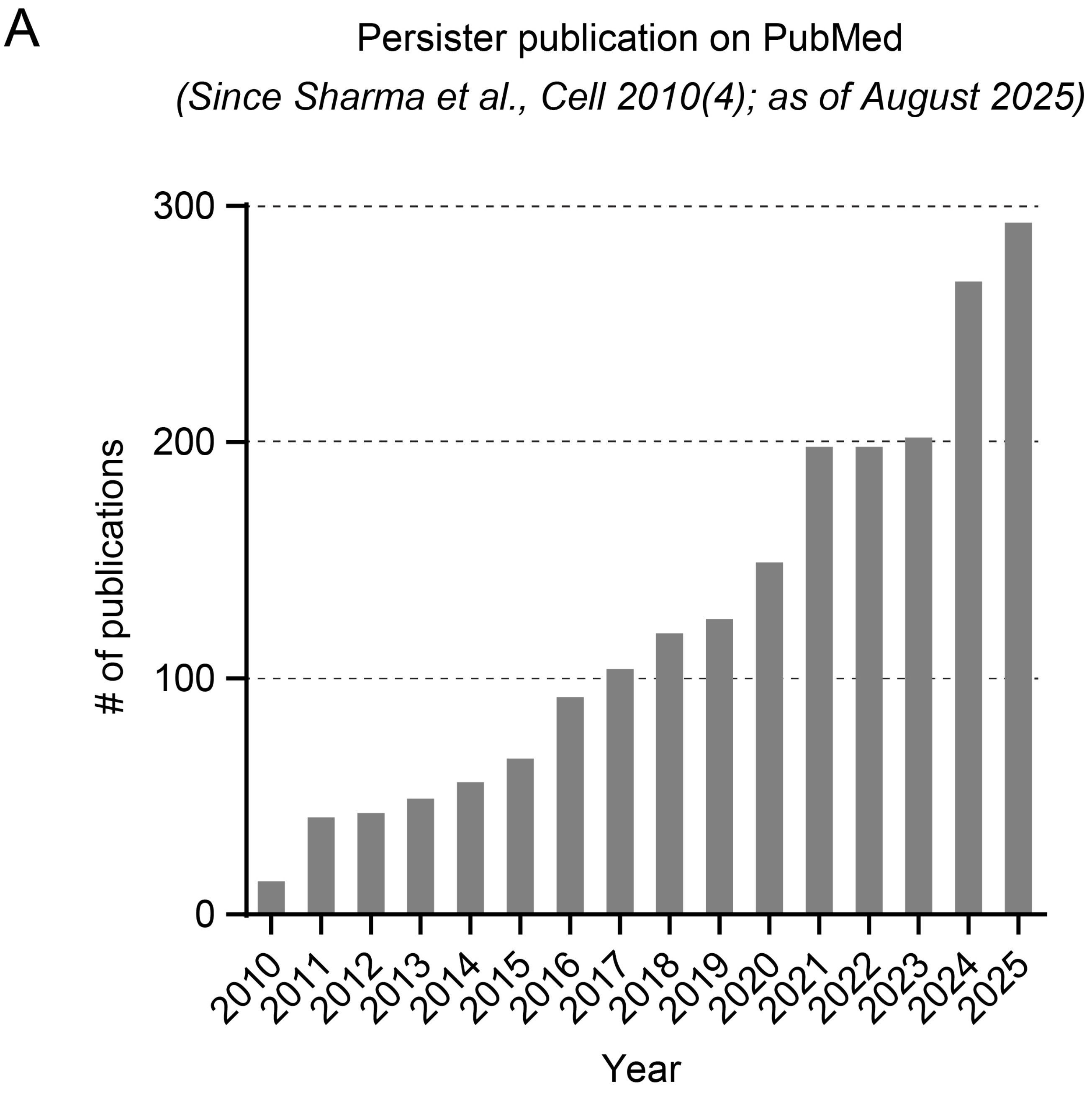

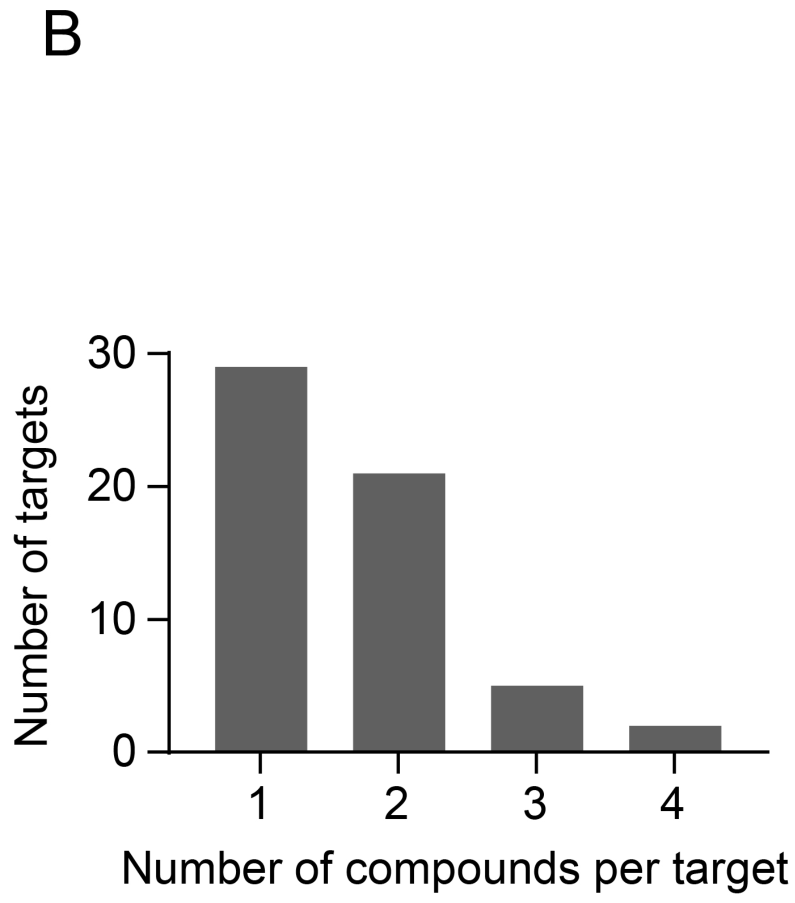

Supplementary Figure S1. Construct the benchmark library for the ResMap screen.

A. Growth of persister-focused publications from 2010 to 2025, illustrating the rapid expansion of the field.

B. The number of compounds available per target in the ResMap benchmark library.

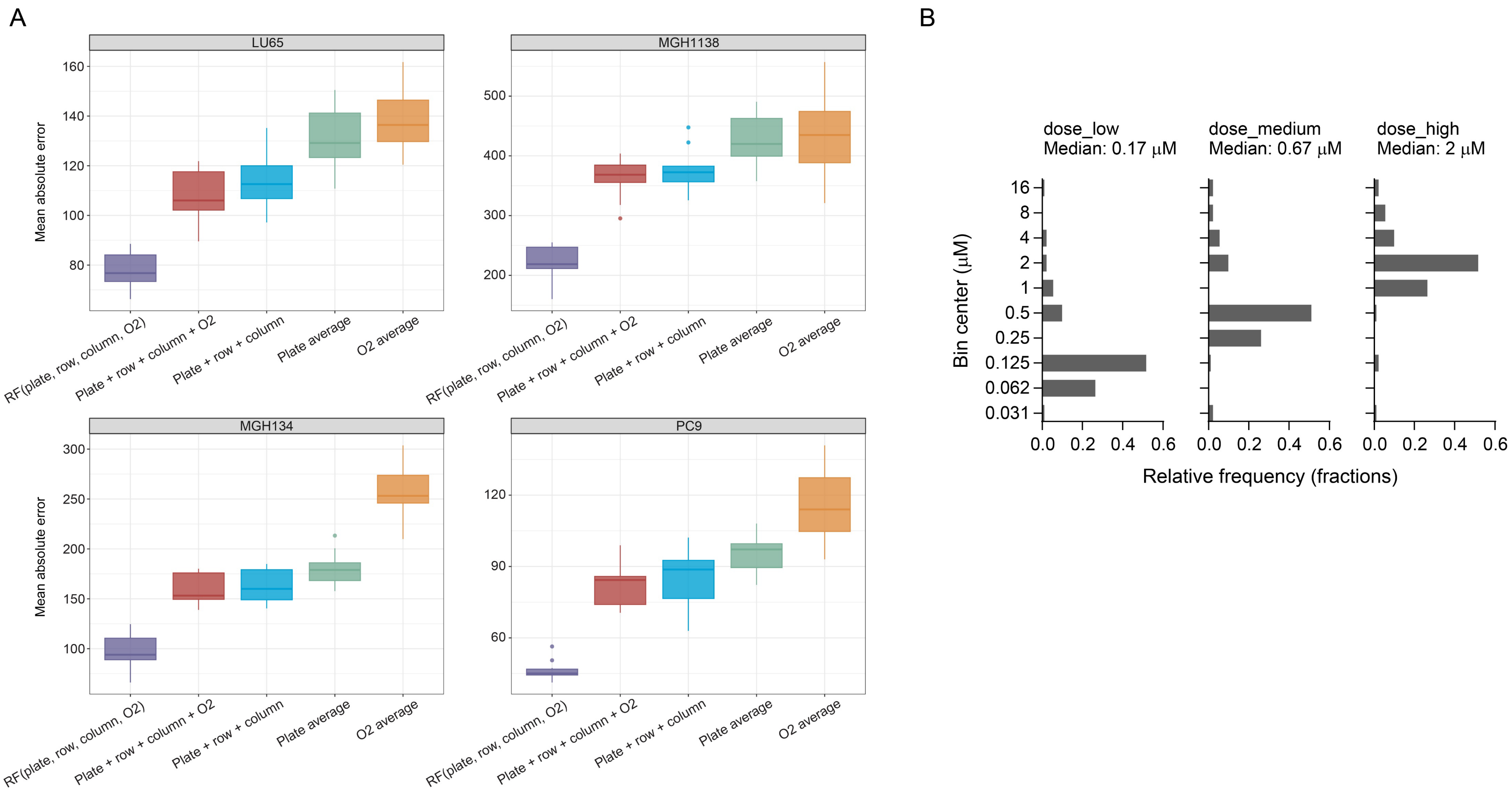

Supplementary Figure S2. Development of the ResMap platform for standardized persister perturbation screening.

A. Model evaluation for normalization. Mean absolute error (MAE) between predicted and observed drug-only control well counts, evaluated on a randomly sampled held-out set (25% of control wells per plate). Color indicates normalization method: purple, random forest model fit on plate ID, well row/column position, O_2 condition; red, linear regression model (same features); blue, linear regression (excluding O_2 condition); green, plate-level average; orange, O_2-level average. Models incorporating technical features and O_2 status reduced prediction error compared to global averages.

B. Distribution of compound doses tested in the ResMap screen. Histogram showing low, medium, and high dosing ranges used across compounds, with median doses of 0.17, 0.67, and 2 μM, respectively.

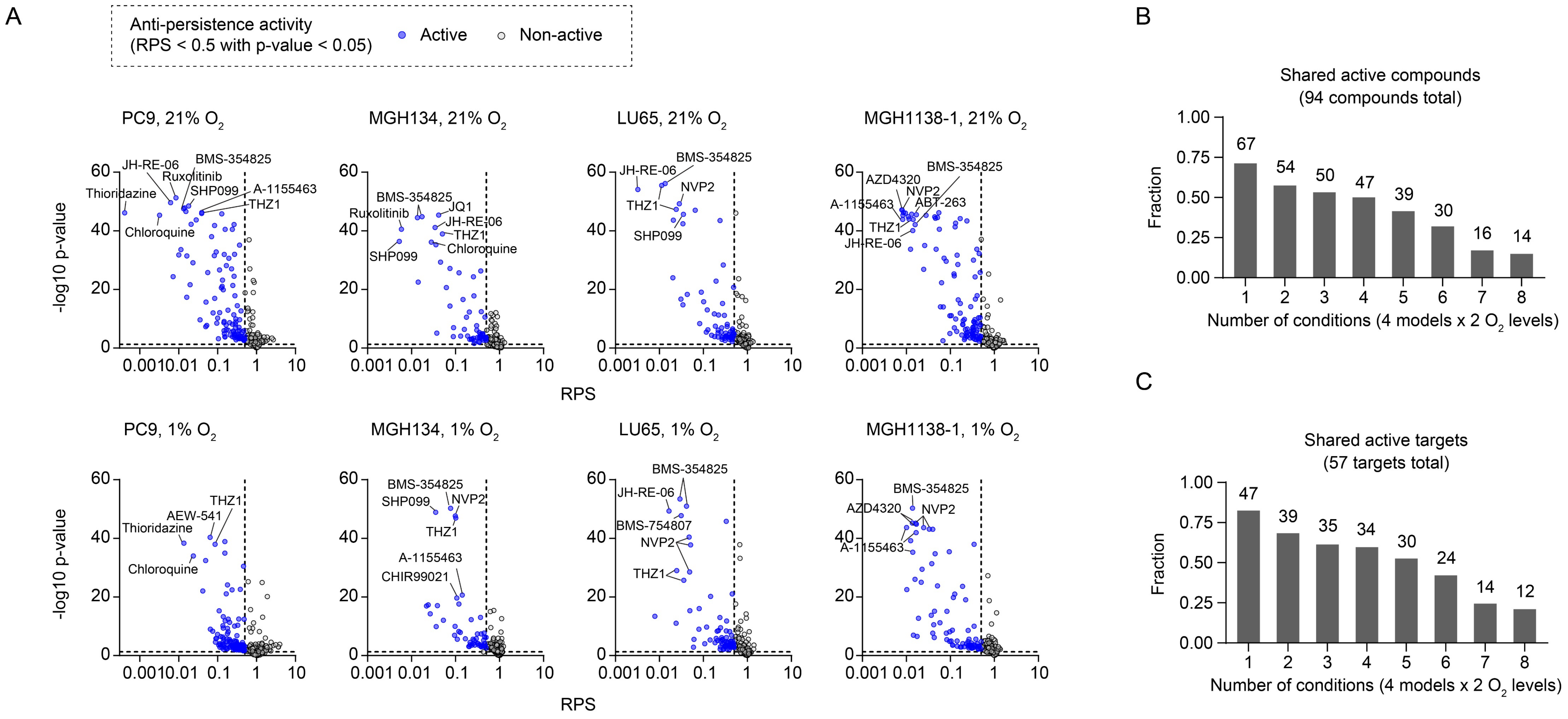

Supplementary Figure S3. Anti-persistence activity in the ResMap screen.

A. Volcano plots summarizing compound-level anti-persistence activity for each model and oxygen condition. Each point represents a compound tested at one dose. Hits (blue) are defined as median RPS < 0.5 (vertical dashed lines) with $p < 0.05$ (horizontal dashed lines); non-active compounds are shown in gray. Representative recurrent actives are labeled.

B-**C.** Fraction of active compounds (**B**) and targets (**C**) shared across the eight model-oxygen conditions. The proportion of actives decreases as more contexts are required for overlap, indicating substantial context specificity of anti-persistence activity.

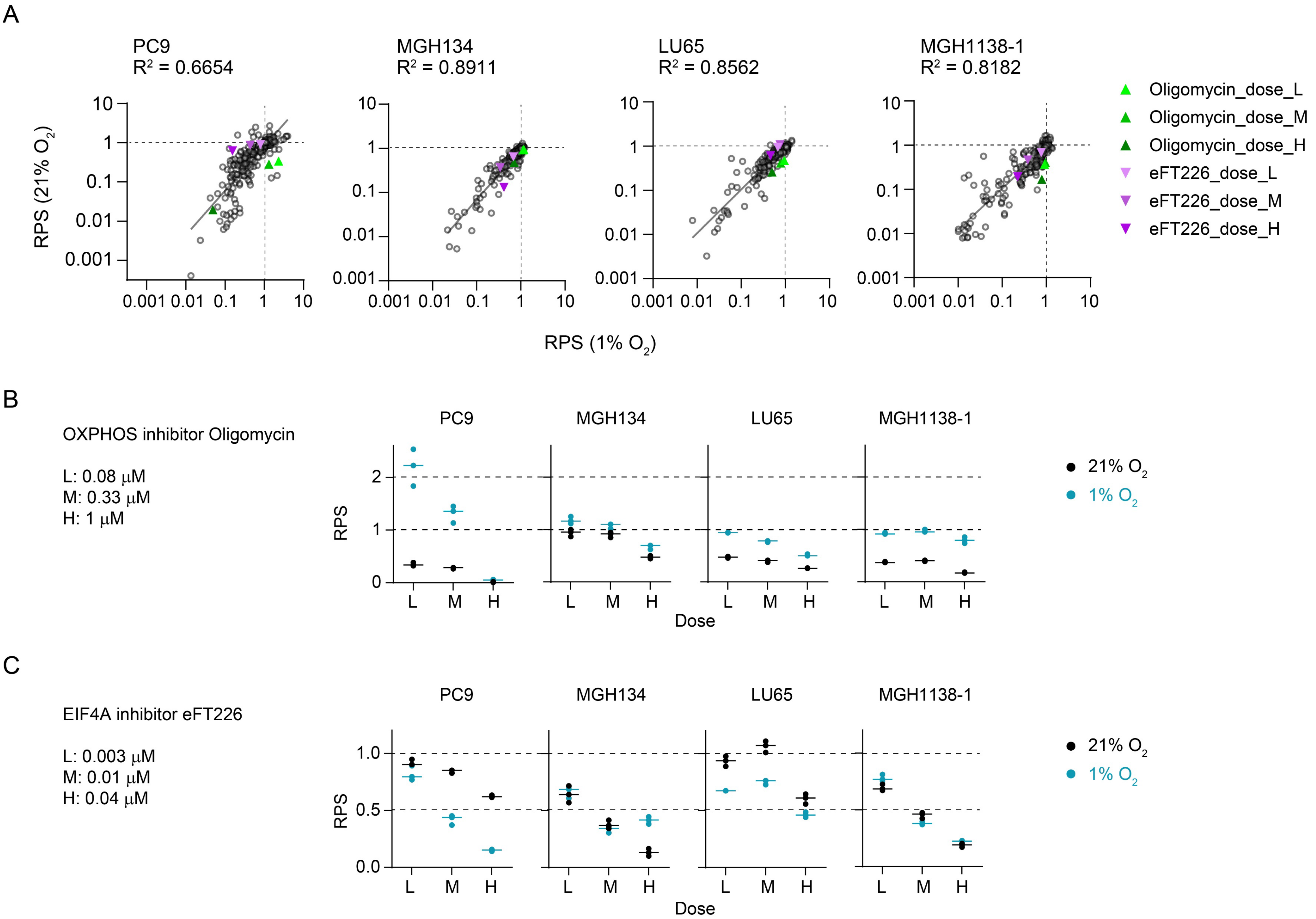

Supplementary Figure S4. Comparison of compound activity between normoxia and hypoxia reveals oxygen-agnostic and oxygen-sensitive responses.

A. Correlation of median RPS values between normoxic (21% O_2) and hypoxic (1% O_2) conditions for each model. Each point represents a compound tested at one dose. Dashed lines indicate baseline persistence (RPS = 1). R^2 values (calculated on the log10 scale) quantify concordance between oxygen levels. Two oxygen-dependent compounds are highlighted: oligomycin (green) and eFT226 (pink).

B. Detailed response of oligomycin, a mitochondrial ATP synthase inhibitor showing stronger anti-persistence activity at 21% O_2 across all models.

C. Detailed response of eFT226, an EIF4A inhibitor showing greater anti-persistence activity at 1% O_2, particularly in PC9 and LU65. Compound activities are shown as triplicate measurements (dots) and median values (solid lines).

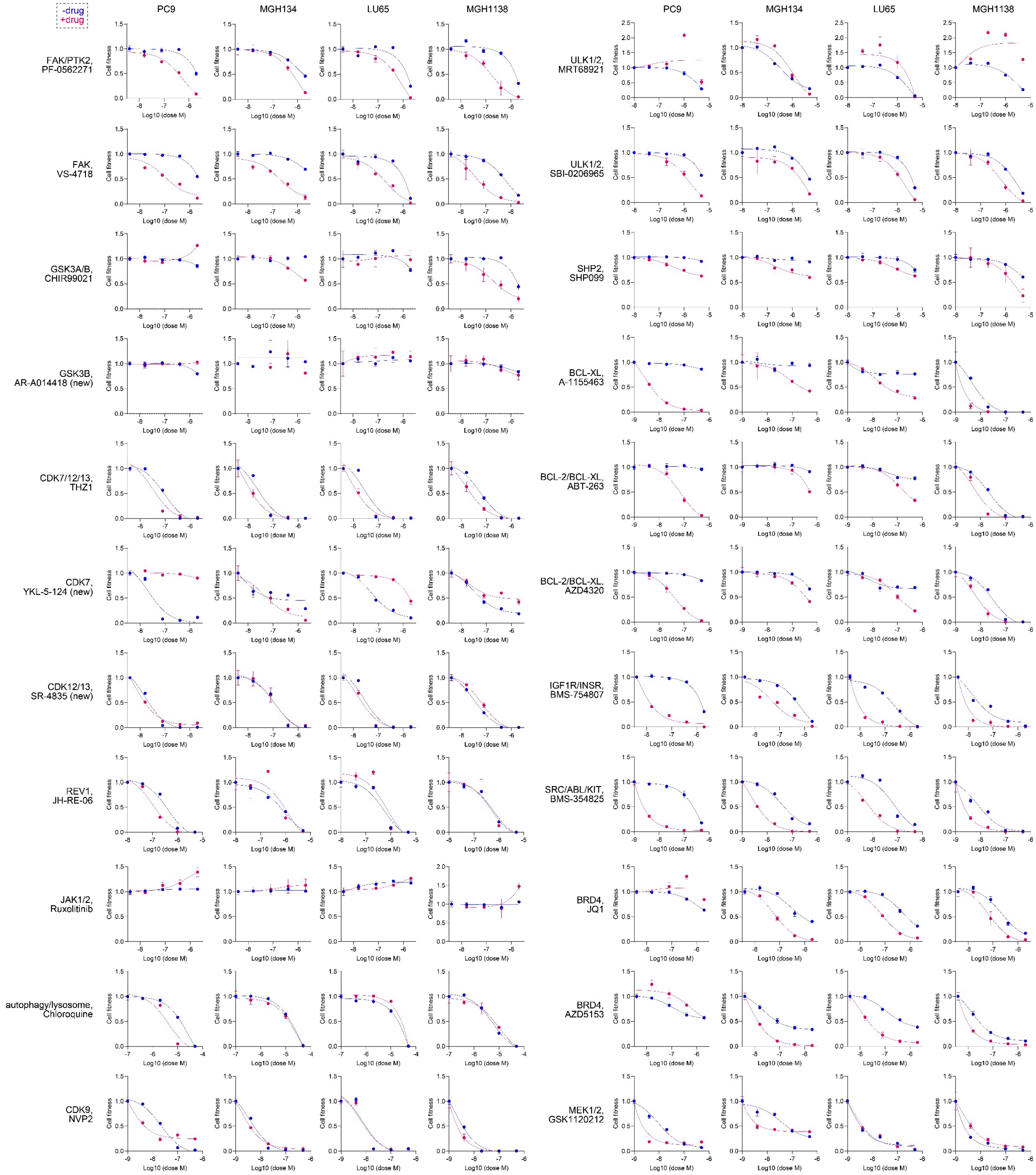

-drug
+drug
PC9
MGH134
LU65
MGH1138
FAK/PTK2, PF-0562271
FAK, VS-4718
GSK3A/B, CHIR99021
GSK3B, AR-A014418 (new)
CDK7/12/13, THZ1
CDK7, YKL-5-124 (new)
CDK12/13, SR-4835 (new)
REV1, JH-RE-06
JAK1/2, Ruxolitinib
autophagy/lysosome, Chloroquine
CDK9, NVP2
ULK1/2, MRT68921
ULK1/2, SBI-0206965
SHP2, SHP099
BCL-XL, A-1155463
BCL-2/BCL-XL, ABT-263
BCL-2/BCL-XL, AZD4320
IGF1R/INSR, BMS-754807
SRC/ABL/KIT, BMS-354825
BRD4, JQ1
BRD4, AZD5153
MEK1/2, GSK1120212
Cell fitness
Log10 (dose M)

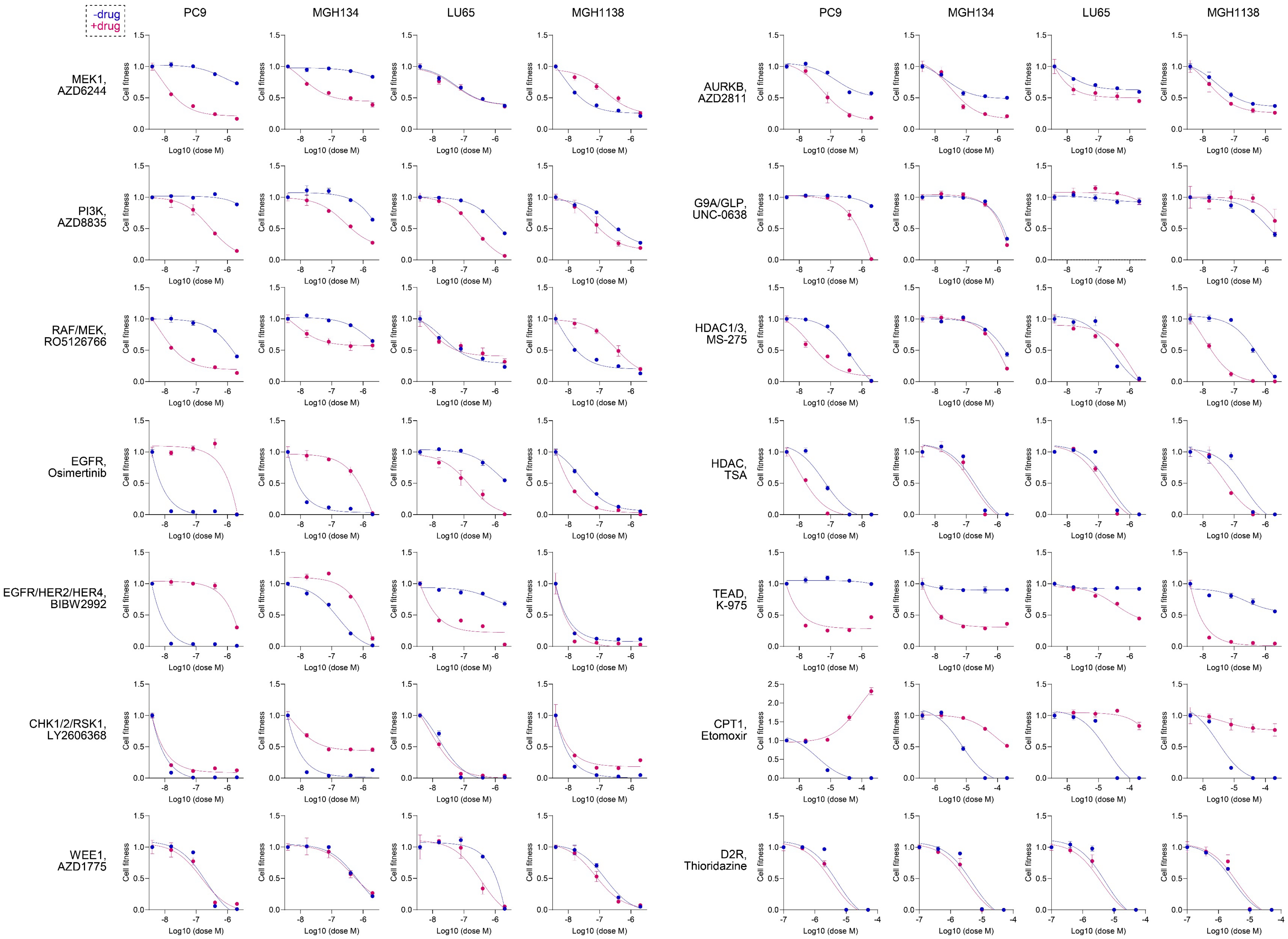

Supplementary Figure S5. Dose-response curves in follow-up validation experiments.

Dose-response curves for all compounds tested in the follow-up validation assay across four lung cancer models under normoxia and ± cancer drug conditions. Target and compound names are listed on the left. Pink curves indicate treatment in the presence of EGFR inhibitor osimertinib (PC9, MGH134) or KRAS inhibitor sotorasib (LU65, MGH1138), and blue curves indicate treatment in the absence of cancer drug. Data are shown as mean of triplicate wells ± standard deviation.

Supplementary Tables

Supplementary Table S1. ResMap compendium of persister targets.

Supplementary Table S2. Composition of compound library for ResMap screen.

Supplementary Table S3. Compound activities from ResMap screen.

Supplementary Table S4. Target-level activities from ResMap screen and original discovery contexts.

Supplementary Table S5. Compound activities conserved across four models from ResMap screen.

Supplementary Table S6. Target-level activities conserved across four models from ResMap screen.

Supplementary Table S7. Follow-up validation of top ResMap screen targets.

Supplementary Table S8. DepMap gene essentiality for top ResMap screen targets.

Supplementary Table S9. MGI gene knockdown/knockout phenotypes associated with top ResMap screen targets.

Supplementary Table S10. MGI gene knockdown/knockout phenotype count for top ResMap screen targets.

Supplementary Table S11. Lung cancer cell lines and culture medium.

Supplementary Table S12. List of reagents.
